# Short term use of an e-cig: influence on clinical symptoms, vital signs and eCO levels

**DOI:** 10.1186/1617-9625-12-S1-A30

**Published:** 2014-06-06

**Authors:** Stamatoula Tsikrika, Sophia Vakali, Sophia Antiopi Gennimata, Anastasios Palamidas, Georgios Kaltsakas, Nikolaos Koulouris, Chritina Gratziou

**Affiliations:** 1 Research unit for Tobacco Control, 1st Respiratory Department, University of Athens, Medical School, Sotiria Hospital, Athens, 11527, Greece

## Background

The risks of electronic cigarette are a subject of uncertainty. The study was designed to assess the acute effect of smoking an e- cigarette on vital signs, clinical symptoms and exhaled markers.

## Materials and methods

Sixty two participants (32 men) with a mean age of 45.43 years have been recruited. Sixteen smokers were suffered by COPD, 12 smokers by asthma, 24 smokers had no overt airways disease. All were current smokers with a long smoking history. A group of 10 non-smokers was also included. The same brand of e-cig was used for 10 min inhaled 11mg. Clinical symptoms, vital signs, - heart rate, oxygen saturation (SpO2) and exhaled CO, was assessed pre and post the e-cig use.

## Results

Cough and sore throat were presented in both groups, of non-smokers and smokers following the e-cig smoking. Sore throat and cough were reported by 90% of asthmatics and 63% of COPD. A significant increase in heart rate (p<0.05) with palpitations was also noted with a decrease in SpO2 mainly smokers (p<0.05). An interesting finding was the significant increase in exhaled CO in the group of non-smokers (p<0.05). Smoking an e-cig was acceptable and gave a feeling of pleasure in a low number of participants (18 % of smokers, 27 % of smokers with asthma and 43% in smokers with COPD). There were also a 12% of non smokers who have easily accepted its use.

## Conclusions

Our study shows that even a single use of an e-cigarette increased heart rate and symptoms like cough and sore throat. Claims that electronic cigarettes can help smokers quit need to be backed up by clinical studies and toxicity analyses and operate within the proper regulatory framework.

